# Photoactivated Localization Microscopy with Bimolecular Fluorescence Complementation (BiFC-PALM) for Nanoscale Imaging of Protein-Protein Interactions in Cells

**DOI:** 10.1371/journal.pone.0100589

**Published:** 2014-06-25

**Authors:** Andrew Nickerson, Tao Huang, Li-Jung Lin, Xiaolin Nan

**Affiliations:** Department of Biomedical Engineering, Knight Cancer Institute, and OHSU Center for Spatial Systems Biomedicine, Oregon Health and Science University, Portland, Oregon, United States of America; Julius-Maximilians-University Würzburg, Germany

## Abstract

Bimolecular fluorescence complementation (BiFC) has been widely used to visualize protein-protein interactions (PPIs) in cells. Until now, however, the resolution of BiFC has been limited by the diffraction of light to ∼250 nm, much larger than the nanometer scale at which PPIs occur or are regulated. Cellular imaging at the nanometer scale has recently been realized with single molecule superresolution imaging techniques such as photoactivated localization microscopy (PALM). Here we have combined BiFC with PALM to visualize PPIs inside cells with nanometer spatial resolution and single molecule sensitivity. We demonstrated that PAmCherry1, a photoactivatable fluorescent protein commonly used for PALM, can be used as a BiFC probe when split between residues 159 and 160 into two fragments. PAmCherry1 BiFC exhibits high specificity and high efficiency even at 37°C in detecting PPIs with virtually no background from spontaneous reconstitution. Moreover, the reconstituted protein maintains the fast photoconversion, high contrast ratio, and single molecule brightness of the parent PAmCherry1, which enables selective PALM localization of PPIs with ∼18 nm spatial precision. With BiFC-PALM, we studied the interactions between the small GTPase Ras and its downstream effector Raf, and clearly observed nanoscale clustering and diffusion of individual KRas G12D/CRaf RBD (Ras-binding domain) complexes on the cell membrane. These observations provided novel insights into the regulation of Ras/Raf interaction at the molecular scale, which would be difficult with other techniques such as conventional BiFC, fluorescence co-localization or FRET.

## Introduction

Protein-protein interactions (PPIs) play a central role in biology[1], yet fundamental information such as their subcellular location is lacking for many of them. Among current techniques for analyzing PPIs, bimolecular fluorescence complementation (BiFC) has been commonly used for its ability to directly visualize PPIs within a cell[2], [3], [4]. In BiFC, a fluorescent protein is split into two non-fluorescent fragments, each genetically fused to a candidate protein of interest. When the two candidate proteins interact, the fragments are brought into proximity to reconstitute a complete fluorescent protein. This allows detection of PPIs with high sensitivity and subcellular resolution. A fundamental limitation of conventional BiFC and light microscopy, however, is that the best spatial resolution is ∼250 nm due to the diffraction of light. This resolution is insufficient considering the scale at which PPIs occur, i.e., a few nanometers. Moreover, as exemplified by lipid rafts[5] and Ras nanoclusters[6], PPIs are often spatially regulated at the nanometer scale. Hence, a clear understanding of PPIs and their roles in cellular processes requires that PPIs be visualized with nanometer resolution.

Several techniques have recently been developed to overcome the resolution limit of fluorescence microscopy, including photoactivated localization microscopy (PALM)[7], [8], stochastic optical reconstruction microscopy (STORM)[9], and their derivatives. PALM and STORM are both based on stochastic switching and subdiffractive localization of individual fluorescent molecules, and routinely offer 10–20 nm spatial resolution in imaging whole biological samples. Stochastic switching of individual fluorescent molecules is achieved by using photoswitchable fluorescent proteins in PALM[7], [8], pairs of organic fluorophores in STORM[9], or standard organic fluorophores in direct STORM (*d*STORM)[10].

We reasoned that, since fluorescent proteins (FPs) are used in both PALM and BiFC, it is possible to combine the two approaches (i.e., BiFC-PALM) to achieve imaging of PPIs in cells with nanometer resolution. Specifically, if a photoswtichable instead of a regular FP is used for BiFC, the reconstituted protein may exhibit photoswitching properties similar to the original protein, which will then allow nanoscale imaging of PPIs with PALM. To date, Dronpa is the only photoswtichable FP that has been split for BiFC[11], but PALM imaging with split Dronpa has not been reported. Dronpa is not ideal for PALM imaging because it emits a small number (<200) of photons per activation cycle and exhibits a low contrast ratio[12].

In this manuscript, we report a BiFC system based on split PAmCherry1 for nanoscale imaging of PPIs with PALM. PAmCherry1 is an mCherry-derived photoactivatable fluorescent protein (PA-FP) with biochemical and photophysical properties suitable for genetic tagging and PALM imaging of cellular proteins[13]. It is a monomeric protein that exhibits relatively fast maturation, moderate single-molecule photon yield, irreversible photoactivation, and a high contrast ratio. Moreover, it has been successfully used in quantitative PALM imaging experiments to measure protein stoichiometry[14]. We first validated PAmCherry1 as a probe for BiFC-PALM and optimized the split site, then used it to investigate the nanoscale heterogeneities of Ras/Raf interactions on the cell membrane. BiFC-PALM allowed us to visualize nanoscale clustering of Ras/Raf complexes and track their diffusion in live cells at the single molecule level. Thus, the use of a PA-FP with BiFC further extends the applicability of both techniques.

## Materials and Methods

### Cloning

We used the In-Fusion HD Cloning kit (639649, Clontech) to generate genetic fusions in the pENTR (Life Technologies) backbone, and the Gateway LR Clonase II kit (11791, Life Technologies) to shuttle the resulting fusion constructs from the entry clones to expression clones. We have used both the pcDNA3 or a lentiviral backbone (pLenti-puro-CMV/TO, 17293, Addgene) for the expression clones. PCR fragments used for In-Fusion reactions were generated using the Phusion High-Fidelity DNA Polymerase (M0530, New England Biolabs). For all fusion constructs used in this study, a flexible (GGGGS)_2_ linker was genetically inserted between the PAmCherry1 fragments and the target protein. In generating the inducible heterodimerization constructs, N-Myr signal and a single DmrA domain were subcloned from pHet-Mem1, and a DmrC domain was subcloned from pHet1, both plasmids in the iDimerize Inducible Heterodimer System (635067, Clontech). KRas G12D and CRaf RBD (residues 51–131) were both subcloned from plasmids used in a previous study[14]. The RBD R89L mutation was introduced through site-directed mutagenesis.

### Cell culture and transfection

U2OS cells (HTB-96, ATCC) were cultured at 37°C and 5% CO_2_ in DMEM supplemented with 10% FBS (11995 and 10082 respectively, Life Technologies). Cells were plated in phenol red-free DMEM (21063, Life Technologies) supplemented with 10% FBS on a #1.5 Lab-Tek chamber slide (155409, Thermo Scientific) for PALM imaging after fixation, or a 0.17 mm coverslip bottom Delta T Dish (04200417, Bioptechs) for live cell imaging. Plasmids for the artificial dimerization system were transiently transfected using X-tremeGENE HP (13873800, Roche) as described by the manufacturer. Dimerization was induced by adding 500 nM A/C Heterodimerizer (635057, Clontech) and incubating at 37°C for 2 hours or overnight as indicated. KRas G12D and CRaf RBD constructs were introduced into the cells by lentiviral infection using the ViraPower packaging system (K497500, Life Technologies). For PALM imaging, cells were fixed in fresh 3.7% PFA with 0.1% glutaraldehyde for 15 minutes at room temperature and changed to imaging buffer (100 mM Tris with 30 mM NaCl and 20 mM MgCl_2_, pH 8.5) after fixation. Gold particles (100 nm, EM.GC100, BBI International) were added as fiducial markers to correct for stage drift during imaging.

### Microscopy and data analysis

PALM imaging and tracking was performed on a Nikon Ti-U inverted microscope equipped with a Nikon 60× APO TIRF objective (NA = 1.49) using µManager[15]. Static PALM images were acquired at room temperature. Total internal reflection (TIR) illumination was used in all PALM imaging experiments. PALM image reconstruction was performed using home-written scripts in MatLab (Mathworks, MA). Ripley's K-test and cluster analysis were described previously[14], [16].

To quantify BiFC signal, multiple (4–10 as indicated in the text) random fields of view each containing a few cells were imaged in epi-fluorescence mode before and immediately after a pulse (∼1 s) of high 405 nm illumination (125 W/cm^2^). Fluorescence from cells with clear photoactivation signals above a threshold was averaged and adjusted for background. For the comparison between wild type CRaf RBD and the R89L mutant, we averaged the fluorescence intensities across the entire field of view because most of the cells with CRaf R89L were dim and had signal levels only slightly above background.

Single molecule tracking experiments were performed at 37°C using a temperature-controlled sample stage (Delta-T, Bioptech). Trajectory analyses were performed using home-written scripts in MatLab (Mathworks, MA). Localizations of molecules in neighboring frames were joined into diffusion trajectories based on spatial proximity, similar to previously reported[17]. Only molecules that lasted at least two frames were used to reconstruct diffusion trajectories. At 50 ms exposure time, we set the maximum distance allowed for a molecule to travel per frame at 500 nm (4 pixels) to avoid falsely connecting localizations of two different molecules into a diffusion trajectory. This is equivalent to a maximum diffusion constant of 1.39 µm^2^/s. Trajectory analysis with variational Bayes single particle tracking (vbSPT) was performed using the MatLab scripts (http://sourceforge.net/projects/vbspt/) provided by the authors [18].

## Results

### BiFC-PALM using PAmCherry1

**Figure 1:**
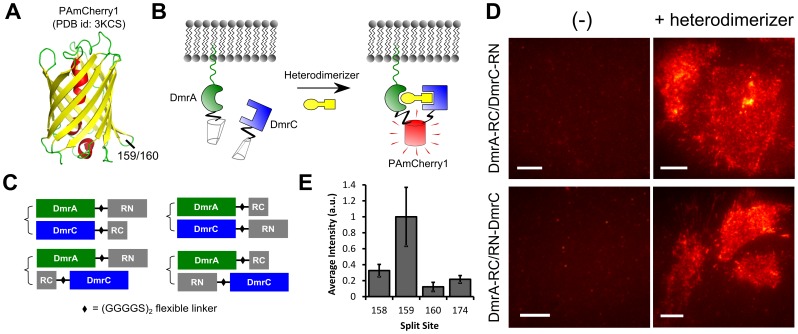
Split PAmCherry1 for BiFC. (A) Crystal structure of PAmCherry1 with the 159/160 split site for BiFC indicated. The site is located between beta sheets 7 and 8; (B) Artificial dimerization system for testing PAmCherry1 BiFC. Non-fluorescent PAmCherry1 fragments are fused to dimerization domains, DmrA and DmrC. The N-terminus of DmrA has a myristoylation signal that localizes it to the plasma membrane. DmrA/DmrC heterodimerization is induced with the addition of a small molecule heterodimerizer, bringing the PAmCherry1 fragments together to reconstitute an intact PAmCherry1; (C) Four configurations were tested for BiFC with the inducible heterodimerization system. A flexible (GGGGS)_2_ linker was used for all constructs. RN  =  PAmCherry1 N-terminal fragment (residues 1–159), RC  =  PAmCherry1 C-terminal fragment (residues 160–236); (D) Total internal reflection fluorescence (TIRF) images of heterodimerizer induced BiFC signals in DmrA-RC/DmrC-RN (top right panel) and DmrA-RC/RN-DmrC (bottom right panel). Constructs were transiently transfected into U2OS cells and cells on the right were exposed to 500 nM heterodimerizer overnight prior to imaging; (E) Average BiFC signal intensities at four different split sites: 158, 159, 160 and 174 (n = 5–10). Intensities are normalized to split site 159. Error bars are SEM. Scale bars, (D) 10 µm.

The efficiency of fluorophore reconstitution in BiFC is sensitive to the position of the split site[4]. In a previous report, Fan *et al.* tested different split sites for mCherry, the parent protein of PAmCherry1, and found that site 159 (i.e., split between residues 159 and 160) was optimal for BiFC[19]. In the crystal structure of PAmCherry1[20], this site is located in the loop between beta-sheets 7 and 8 (Fig.1A), a position successfully used for BiFC of many FPs[4]. PAmCherry1 and mCherry also have identical amino acid sequences around residues 159 and 160 (Fig. S1). Based on these factors, we hypothesized that site 159 would be suitable for PAmCherry1 BiFC.

To test this hypothesis, we genetically fused the two fragments, RN (residues 1–159) and RC (residues 160–236), to DmrA and DmrC, two peptide components from the Clontech iDimerize Inducible Heterodimer System (Fig. 1B). DmrA and DmrC do not spontaneously interact but will form a 1∶1 complex when a small molecule heterodimer is added. The DmrA peptide has an N-terminal myristoylation signal for membrane localization, so RN or RC fragments were only fused to its C-terminus. This left us with four possible fusion configurations for BiFC: DmrA-RN/DmrC-RC, DmrA-RN/RC-DmrC, DmrA-RC/DmrC-RN, and DmrA-RC/RN-DmrC (Fig. 1C). A flexible polypeptide linker, (GGGGS)_2_, was used in all fusion constructs[21].

Two of the four configurations, DmrA-RC/DmrC-RN and DmrA-RC/RN-DmrC, yielded strong BiFC signal when expressed in cells and treated with the heterodimerizer (Fig. 1D, right panels, and Fig. S2). As a control, we observed virtually no BiFC signal in the absence of the heterodimerizer (Fig. 1D, left panels) suggesting that RN and RC fragments do not spontaneously interact and reconstitute PAmCherry1 under these conditions. Similar results were obtained when using cytosolic DmrA and DmrC (data not shown), indicating that the lack of spontaneous interaction between RN and RC was not due to the difference in subcellular localizations. Upon addition of the heterodimerizer, significant BiFC signal was developed in 1–2 hours (Fig. S3), which is typical for the time required for chromophore maturation. Similar to other BiFC systems[2], [3], [4], PAmCherry1 BiFC appeared to be irreversible; that is, once PAmCherry1 is reconstituted, it does not disassemble into RN and RC (Fig. S3). This can at times limit the use of BiFC. Interestingly, PAmCherry1 fluorophore reconstituted efficiently at 37°C without cold incubation. By contrast, many other BiFC systems require low temperatures (often at 4°C or 25°C overnight) to function properly[4], which can be detrimental to the cells.

To optimize PAmCherry1 BiFC, we also moved the split site by 1 residue upstream (to site 158) and downstream (to site 160). Sites 158 and 160 only have a partial overlap with the loop between beta-sheets 7 and 8 (Fig. 1A). Using the same DmrA-RC/RN-DmrC configuration, we observed much lower BiFC signals with both site 158 and 160 compared with 159 (Fig. 1E). Similarly, site 174 (split between amino acids 174 and 175) was also much less efficient than site 159 in both mCherry[19] and PAmCherry BiFC (Fig. 1E). These results suggest that site 159 is optimal for PAmCherry1 BiFC. We therefore used fragments RN(1–159) and RC(160–236) in all subsequent experiments.

**Figure 2:**
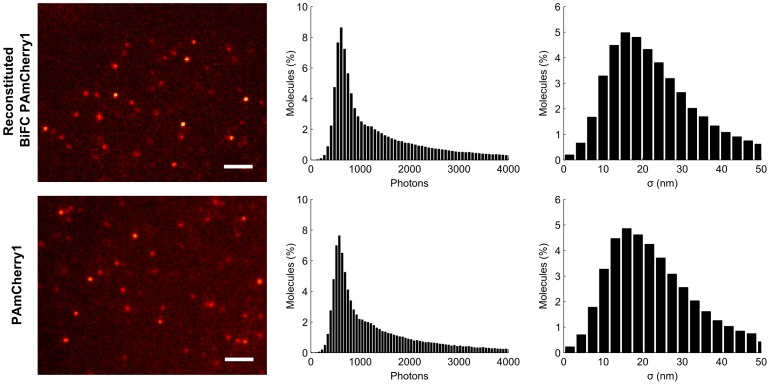
Reconstituted PAmCherry1 shows similar photophysical properties as the original PAmCherry1. U2OS cell expressing RN-KRas G12D and CRaf RBD-RC were imaged next to cells expressing KRas G12D fused to PAmCherry1. Examples of single molecule images are shown with photon and localization precision distributions (n = 5). Scale bars, 2 µm.

BiFC-reconstituted PAmCherry1 possesses similar photophysical properties to the original PAmCherry1, likely because their chromophore structures are nearly identical. In particular, we observed fast photoactivation and a high contrast ratio on BiFC-positive cells (Video S1). Using low power 405 nm activation (5–10 W/cm^2^), single molecule images of reconstituted PAmCherry1 were easily obtained, showing comparable brightness and signal to background ratio with the original PAmCherry1 (Fig. 2). Taken side by side, both emitted ∼600 photons per localization event and achieved ∼18 nm localization precision on average, which are comparable to literature values[13]. Moreover, using a method developed by Annibale *et al* [22], we measured the dark state life time of BiFC-reconstituted PAmCherry1 as 0.26±0.05 *s* (Fig. S4), identical to that of the parent PAmCherry1[14]. These similarities allowed us to perform PALM imaging on PAmCherry1 BiFC samples to obtain nanoscale spatial maps of the DmrA/DmrC artificial complexes (Fig. S5) in fixed cells.

### Visualize Ras/Raf Interactions with Nanometer Resolution using BiFC-PALM

With the principle of BiFC-PALM demonstrated, we next used the technique to study the interactions between Ras and Raf, two important proteins in normal and tumor cell signaling. When Ras is activated by upstream stimuli or by mutations, it recruits and activates Raf at the cell membrane[23]. Recent studies indicated that nanoscale clustering is critical to Ras-mediated activation of Raf[6], [14]. Specifically, Ras has been shown to form 5–8 membered clusters on the cell membrane with typical diameters around 20 nm; this clustering behavior appeared to be driven mostly by the C-terminal CAAX motif of the Ras protein[6], [24]. Ras nanoclusters may serve as signaling platforms to recruit and activate downstream effectors such as Raf. Interestingly, recent studies showed that Raf also signals as dimers and/or multimers mediated through interactions between the kinase domain, for example in the presence of mutant KRas or when the N-terminal domain is truncated[25], [26], [27]. These observations raise the possibility that a functional Ras/Raf signaling assembly may contain multiple (e.g. two) copies of a Ras/Raf complex. Existence of higher order Ras/Raf complex structures, however, has not been directly confirmed. We addressed this question by using BiFC-PALM to reveal the nanoscale spatial organization of individual Ras/Raf complexes on the membrane of fixed cells and diffusion dynamics in living cells.

**Figure 3:**
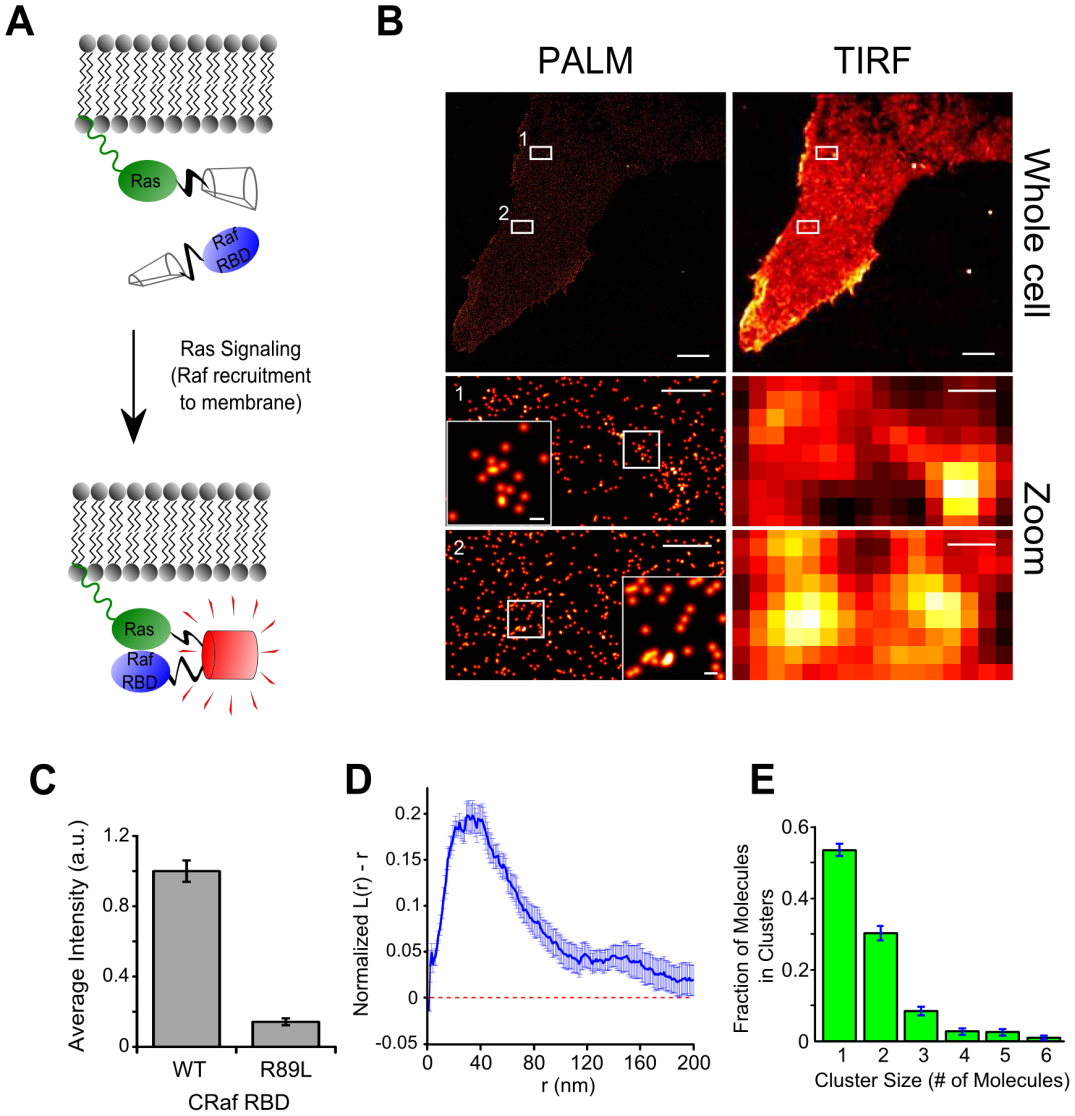
BiFC-PALM imaging and analysis of individual KRas G12D/CRaf RBD complexes. (A) For BiFC, KRas G12D and CRaf RBD (CRaf residues 51–131) were fused to PAmCherry1 fragments. Interaction between CRaf RBD and active KRas G12D on the membrane brings the two fragments into proximity and reconstitute PAmCherry1; (B) PALM images (left) and low-resolution TIRF representations (right) of a U2OS cell expressing RN-KRas G12D and CRaf RBD-RC. Bottom panels are zoomed-in views of boxed areas 1 and 2 in the whole cell views, respectively. Even higher magnification views of boxed subareas of 1 and 2 are displayed as insets in the respective bottom corners; (C) Average BiFC signal intensity with wild type CRaf RBD and the R89L mutant (n = 5−8); (D) Ripley's K-test analysis of ten sampled areas from the cell in B. Dashed line represents random monomeric distribution; (E) Average cluster size distribution within the same ten sampled areas as D. All error bars are SEM in (C), SD in (D) and (E). Scale bars in (B), 5 µm top, 500 nm middle and bottom, 50 nm insets.

We designed a BiFC-PALM system by fusing RN to the N-terminus of KRas 4B (hereafter referred to as ‘KRas’) G12D, an active mutant of KRas, and RC to the C-terminus of the Ras-binding domain (RBD[28], amino acids 51–131) of CRaf (Fig. 3A). Consistent with the strong interactions between KRas G12D and CRaf, the BiFC system yielded a clear signal when expressed in cells (Fig. 3B; top panel). Interestingly, two other KRas G12D/CRaf RBD PAmCherry1 BiFC configurations that we tested were also positive (Fig. S6, although one of the two configurations showed much lower efficiency). As a control, we introduced the R89L point mutation to CRaf RBD to disrupt Ras/Raf interaction[29] and observed that the BiFC signal decreased by nearly 10-fold (Fig. 3C and Fig. S7). These data confirm that the BiFC signal was due to specific interactions between KRas G12D and CRaf RBD.

We imaged the KRas G12D/CRaf RBD complexes in fixed cells with PALM under total internal reflection (TIR) conditions. When reconstructing the PALM images, we corrected blinking (due to transient dark states) of individual PAmCherry1 molecules using a previously described approach[14], [22] and a dark state life time of 0.26 *s*. As such, each dot in the final high resolution image represents a single PAmCherry1-tagged KRas G12D/CRaf RBD complex. An example BiFC-PALM image of PAmCherry1-KRas G12D/CRaf RBD is shown in Fig. 3B (left panels). From the PALM images, it became evident that KRas G12D/CRaf RBD complexes are heterogeneously distributed on the cell membrane as both monomers and clusters (Fig. 3B; middle and bottom left panels, and insets). Spatial pattern analysis with Ripley's K-test revealed that the clusters have an apparent diameter of ∼30 nm (Fig. 3D). We note that this apparent diameter reflects mostly the actual resolution achieved in our PALM imaging experiments and not necessarily the actual size of the clusters. Analysis with simulation aided DBSCAN (SAD)[14] further showed that each cluster typically contains 2–3 KRas G12D/CRaf RBD complexes (Fig. 3E). These observations suggest that multiple Ras/Raf complexes can cluster and form a higher order assembly.

**Figure 4:**
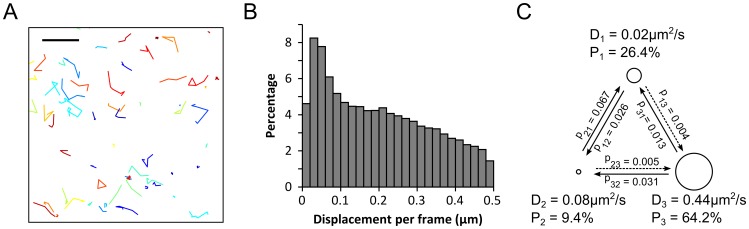
Single-molecule tracking of individual KRas G12D/CRaf RBD complexes. (A) Displacement trajectories of PAmCherry1-tagged KRas G12D/CRaf RBD BiFC complexes showcasing immobile molecules (small trajectory areas) and mobile molecules (large trajectory areas) from a single cell at 50 ms time resolution; (B) Histogram shows the distribution of displacement per frame extracted from the molecular diffusion trajectories at 50 ms time resolution; (C) Variational Bayes single particle tracking (vbSPT) analysis of the diffusion trajectories showing three putative diffusion states. P values indicate transition probabilities between the states per frame acquisition time (50 ms). Scale bar in (A), 1 µm.

Next, we performed single molecule tracking (smt-) PALM measurements in living cells[17] expressing the RN-KRas G12D/CRaf RBD-RC BiFC pair. Under TIR illumination and low dose (2.5–5 W/cm^2^) 405 nm activation conditions, smt-PALM allowed us to sparsely activate and individually track a small number of PAmCherry1 BiFC tagged KRas G12D/CRaf RBD complexes at a time. We typically acquire ∼10,000 diffusion trajectories (localization events that span 2 frames or more) per cell with 50 ms time resolution (Video S2 and Fig. S8). Analysis of the diffusion trajectories from a single cell revealed that the KRas G12D/CRaf RBD complexes exist in at least two states: a mobile state and an immobile state, which is evident in both the positional trajectories (Fig. 4A) and the histogram of displacement per frame (Fig.4B). Here, the displacement per frame represents the instantaneous diffusion rate of the molecules per unit time (50 ms). The existence of multiple diffusion states of the KRas G12D/CRaf RBD complexes was also confirmed by variational Bayes single particle tracking (vbSPT), a new algorithm developed for analyzing single molecule diffusion trajectories[18]. Specifically, vbSPT showed that the molecules have three diffusion states with diffusion constants of 0.44, 0.08, and 0.02 µm^2^/s (Fig. 4C). This observed heterogeneous diffusion behavior is consistent with the co-existence of KRas G12D/CRaf RBD monomers and multimers (Fig. 3B) as well as heterogeneities in the membrane composition [5].

## Discussion

Recent advances in ‘omics’ measurement technologies have identified the proteins and other components that comprise biological systems. The understanding of how proteins interact with each other and with other components, however, has largely lagged behind. Key to accelerating this process is a set of tools that can probe molecular interactions *in situ* with high sensitivity and sufficient temporal and spatial resolutions.

Several techniques, including BiFC[2], [3], [4], fluorescence resonance energy transfer (FRET)[30], and fluorescence co-localization have been commonly used for visualizing molecular interactions, including PPIs, in whole cells. The advantage of BiFC is that only interacting protein pairs would give rise to the signal, allowing sensitive detection of PPIs even in the presence of a large pool of non-interacting proteins. The strong signals from reconstituted fluorophores also permits spatial mapping of the PPIs with subcellular resolution. Additionally, BiFC only uses one fluorophore, which simplifies the experiment and potentially allows BiFC to be used in conjunction with fluorescence co-localization or FRET to visualize multiple PPIs and their relationships on a single sample.

In the present work, we extended the utility of BiFC by combining it with PALM, a recent single molecule superresolution microscopy technique that enables cellular imaging with nanometer spatial and single copy stoichiometric resolutions[7]. Using PAmCherry1 as the fluorescent probe, we demonstrated that the reconstituted fluorescent protein retained the photophysical properties of the original protein that are important for PALM, such as fast photoactivation, high contrast ratio and relatively high photon yield. As a result, BiFC-PALM allowed us to obtain spatial maps of PPIs in whole cells with ∼18 nm localization precision and single molecule counting capability[14].

Several properties of the PAmCherry1 BiFC system make it suitable for detecting PPIs at physiological conditions. First, it exhibited low background from nonspecific fluorophore reconstitution, similar to its parent protein mCherry[19]. Second, the reconstitution of PAmCherry1 is highly efficient at physiological temperature (37°C). In contrast, many other split FPs including mCherry need to be reconstituted at 25 or 4°C [19]. Despite the similarity between mCherry and PAmCherry1 in structure and sequence, we assume that the minor differences account for the stability of PAmCherry1 BiFC at 37°C.

The main disadvantage of BiFC is that the fluorophore reconstitution process is almost always irreversible once fully formed; this is also true for PAmCherry1. This at times raises the question as to whether the observations authentically reflect the underlying physiological process. Despite this concern, numerous studies have demonstrated the value of BiFC in detecting PPIs inside cells where BiFC signals accurately reported the spatial and temporal characteristics of the process being studied[3], [4].

Besides PAmCherry1, many other PA-FPs that have been used for PALM imaging, such as mEos3[31] (Yujie Sun *et al.* personal communications), PA-GFP[32], Dronpa[33], could also be used for BiFC-PALM. In particular, PA-GFP emits in the green channel and has been successfully used in dual color PALM imaging experiments; it may be split similarly to GFP[3], [4] and used in conjunction with PAmCherry1 in dual-color BiFC-PALM imaging experiments to study nanoscale co-localizations between two different PPIs.

With BiFC-PALM, we were able to show that Ras/Raf complexes can form higher order structures (mostly dimers and trimers, Figs. 3B and E). The results are similar to previous observations on Ras[6] or Raf[14] alone. The observed higher order structures are consistent with the single molecule tracking experiments that revealed heterogeneous populations of Ras/Raf complexes (Fig. 4). Hence, our BiFC-PALM imaging results are consistent with previous hypothesis that a functional Ras/Raf assembly could be a cluster of Ras/Raf protein complexes. Notably, here we have only used the RBD of CRaf, which lacks the kinase domain critical for Raf/Raf dimerization, and still observed the clustering of KRas G12D/CRaf RBD protein complexes. This indicates that the clustering of KRas G12D/CRaf RBD is driven by KRas G12D. Nevertheless, the irreversibility of PAmCherry1 BiFC requires that more experiments are needed to fully elucidate the existence and biological relevance of these higher order Ras/Raf structures.

In summary, we have shown that by using a PA-FP such as PAmCherry1 as a BiFC probe, it is feasible to study the nanoscale spatial organization of interacting protein complexes and track their motion in living cells based on PALM. We anticipate that more PA-FPs will be used for BiFC-PALM, which will open up many new possibilities for studying PPIs in cells with high spatial resolution and at the single molecule level.

## Supporting Information

Figure S1 **Sequence alignment between mCherry and PAmCherry1.** (a) Partial amino acid alignment with residues 159/160 boxed; (b) Graphical comparison of the nucleotide sequences with the codon for residue 159 marked in green.
(TIF)

Figure S2 **Four test configurations for PAmCherry1 BiFC with DmrA/DmrC.** Plasmid combinations as indicated in the four panels were transiently transfected into U2OS cells. After 24 hours, the cells were incubated in 500 nM heterodimerizer overnight, then washed and fixed for imaging. DmrA and DmrC are dimerizing domains, RN  =  PAmCherry1 N-terminal residues 1–159, RC  =  PAmCherry1 C-terminal residues 160–236. All images were acquired in TIRF mode with moderate 405 nm laser illumination.
(TIF)

Figure S3 **Testing the reversibility of PAmCherry1 BiFC.** U2OS cells transiently transfected with DmrA-RC and RN-DmrC were treated with heterodimerizer for 2 hours at 37°C prior to imaging. Cells in one chamber (left) were fixed immediately, and those in another chamber (middle) were incubated for another 2 hours in growth media at 37°C without the heterodimerizer before fixation. No significant difference was observed in BiFC signal intensities between the two samples (right, n = 3).
(TIF)

Figure S4 **Estimating the dark state life time (** ***T_off_*** **) of BiFC-PAmCherry1.** We use a similar approach as described in Annibale *et al.* (ref 22) to estimate the *T*_off_ of PAmCherry1 reconstituted by BiFC. Briefly, the total number of molecules in the final reconstructed PALM image is a function of maximum allowed dark period (*T*_d_). The greater *T*_d_ is the smaller number of molecules remain in the final PALM image because more localization events are combined despite that they are separated by dark periods. This is reflected in the blue curve, where an initial, sharp decrease in the remaining fraction of molecules is followed by a second, slower decrease. The initial phase of the decrease is primarily due to the correction of molecular blinking, i.e., the molecules transiently entering dark states. As *T*_d_ becomes much larger than *T_off_*, emission events from different molecules residing in the same pixel start to get combined resulting in further decrease in the number of molecules. We found that the first 7 points (*T*_d_ up to ∼1 *s*) gave the best fit to a single exponential (R = 0.996); from this fitting (red curve), we obtained *T*_off_ ∼0.26±0.05 *s*.
(TIF)

Figure S5 **Superresolution imaging of DmrA/DmrC complex with BiFC-PALM.** The BiFC configuration used was DmrA-RC/RN-DmrC, where RN and RC are PAmCherry1 fragments split at site 159. (a) PALM image of a cell expressing the BiFC pair; (b) Zoomed-in view of the boxed area in (a); (c) Low-resolution representation of (b).
(TIF)

Figure S6 **PAmCherry1 BiFC to visualize KRas G12D/CRaf RBD interaction.** TIRF images for the four BiFC configurations between KRas G12D and CRaf RBD, each fused with PAmCherry1 fragments RN or RC (split at site 159). U2OS cell lines stably expressing RN-KRas G12D (top panels) or RC-KRas G12D (bottom panels) were generated. RC-CRaf RBD or CRaf RBD-RC was then introduced into either cell line via lentiviral infection ∼24 hours prior to imaging. RN-KRas G12D/CRaf RBD-RC (upper right) was used for Fig. 3B, D and E.
(TIF)

Figure S7 **Effect of the RBD R89L mutation on BiFC of RN-KRas G12D and CRaf RBD-RC.** U2OS cells stably expressing RN-KRas G12D were infected with lentivirus bearing wildtype CRaf RBD-RC (left) or the CRaf RBD R89L-RC mutant (right) and fixed ∼24 hours post infection.
(TIF)

Figure S8 **Single molecule tracking of individual KRas G12D/CRaf RBD complexes.** Live U2OS cells expressing RN-KRas G12D and CRaf RBD-RC were imaged with smt-PALM at 37°C and 50 ms time resolution. Individual molecules were localized and the diffusion trajectories were inferred from the locations of the same molecule in consecutive frames. The trajectories are randomly color-coded for easy distinction.
(TIF)

Table S1 PCR Primers used in this study.
(DOCX)

Video S1 **BiFC-reconstituted PAmCherry1 showing fast photoconversion and high contrast ratio.** Fixed U2OS cells with BiFC-reconstituted PAmCherry1 were imaged with a 561 nm laser under low power 405 nm activation (2.5–10 W/cm^2^).
(AVI)

Video S2 **Live cell smt-PALM.** Live U2OS cells expressing RN-KRas G12D and CRaf RBD-RC were imaged at 37°C and 50 ms time resolution with a 561 nm laser under low power 405 nm activation (2.5 W/cm^2^). Circles indicate molecules being tracked.
(AVI)
