# Supplementary figures and images for: Photoactivated Localization Microscopy with Bimolecular Fluorescence Complementation (BiFC-PALM) for Nanoscale Imaging of Protein-Protein Interactions in Cells
Source: PLoS One. 2014 Jun 25;9(6):e100589. doi: 10.1371/journal.pone.0100589 (PMC4070983; doi:10.1371/journal.pone.0100589)

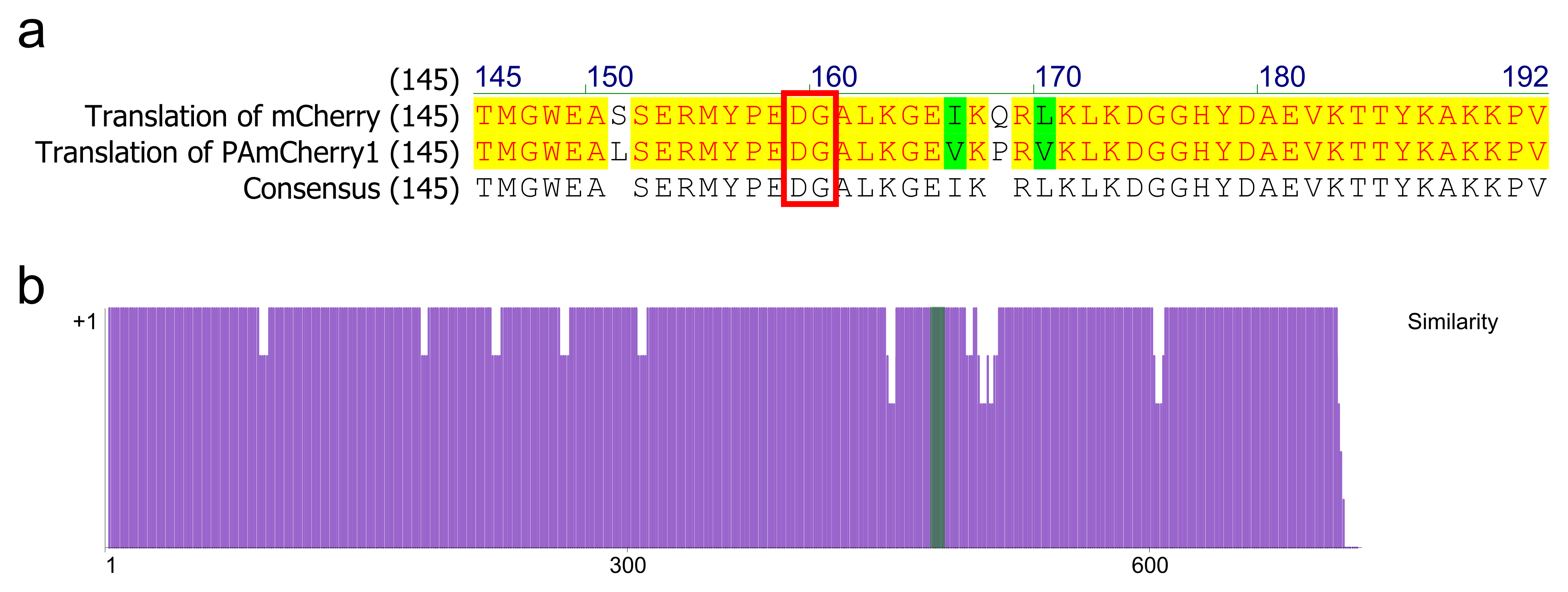

Supplement: Figure S1 — Sequence alignment between mCherry and PAmCherry1. (a) Partial amino acid alignment with residues 159/160 boxed; (b) Graphical comparison of the nucleotide sequences with the codon for residue 159 marked in green. (TIF) [file pone.0100589.s001.tif]

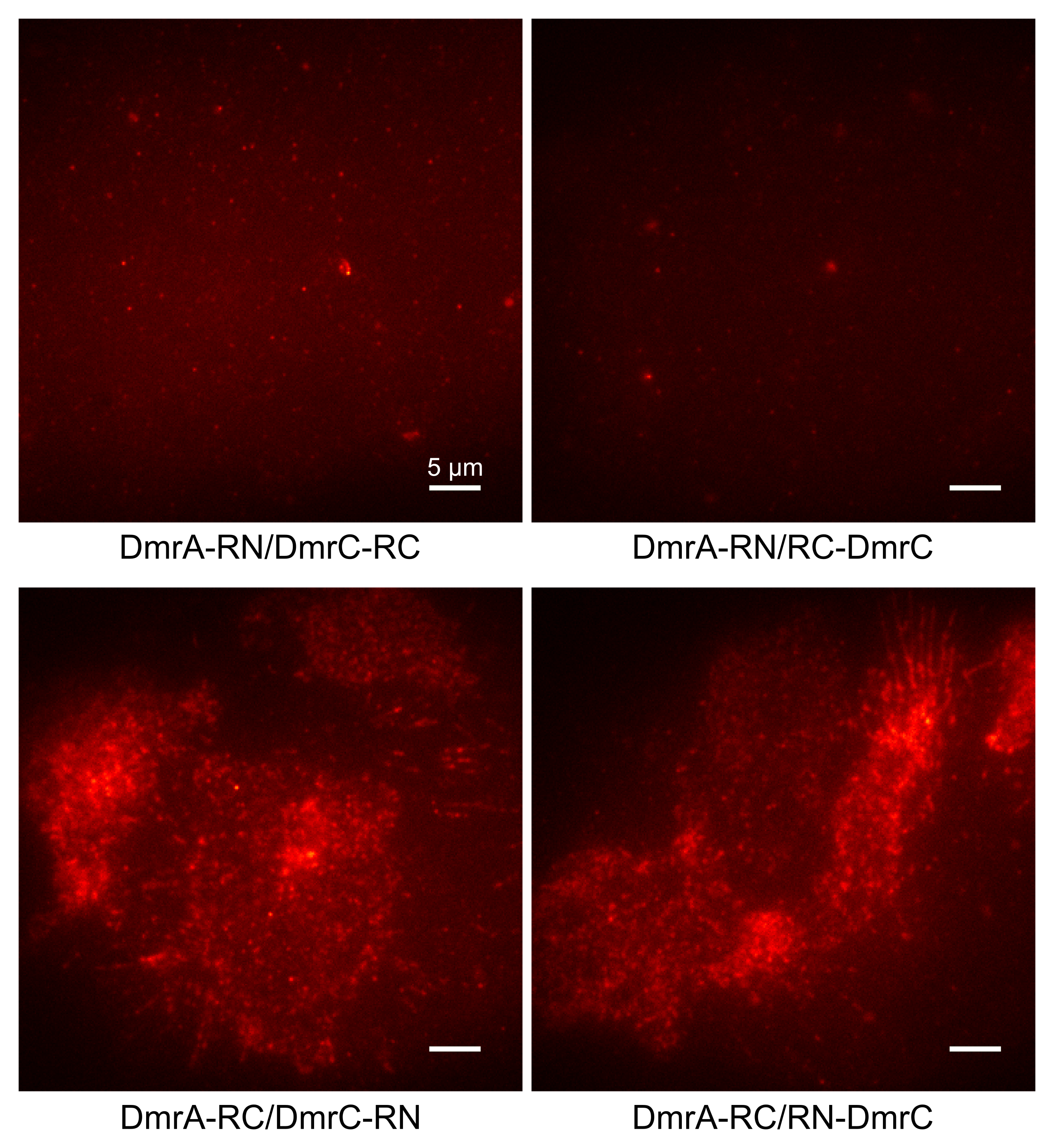

Supplement: Figure S2 — Four test configurations for PAmCherry1 BiFC with DmrA/DmrC. Plasmid combinations as indicated in the four panels were transiently transfected into U2OS cells. After 24 hours, the cells were incubated in 500 nM heterodimerizer overnight, then washed and fixed for imaging. DmrA and DmrC are dimerizing domains, RN = PAmCherry1 N-terminal residues 1–159, RC = PAmCherry1 C-terminal residues 160–236. All images were acquired in TIRF mode with moderate 405 nm laser illumination. (TIF) [file pone.0100589.s002.tif]

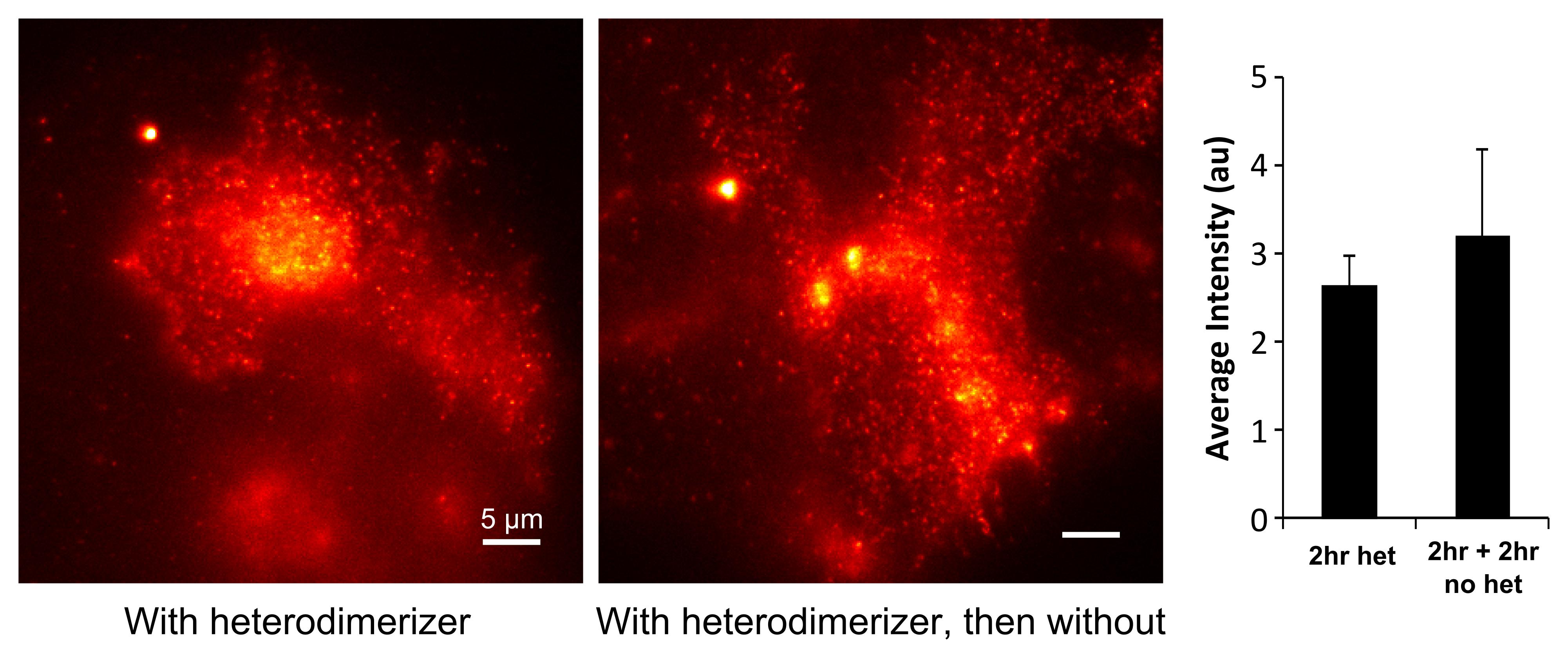

Supplement: Figure S3 — Testing the reversibility of PAmCherry1 BiFC. U2OS cells transiently transfected with DmrA-RC and RN-DmrC were treated with heterodimerizer for 2 hours at 37°C prior to imaging. Cells in one chamber (left) were fixed immediately, and those in another chamber (middle) were incubated for another 2 hours in growth media at 37°C without the heterodimerizer before fixation. No significant difference was observed in BiFC signal intensities between the two samples (right, n = 3). (TIF) [file pone.0100589.s003.tif]

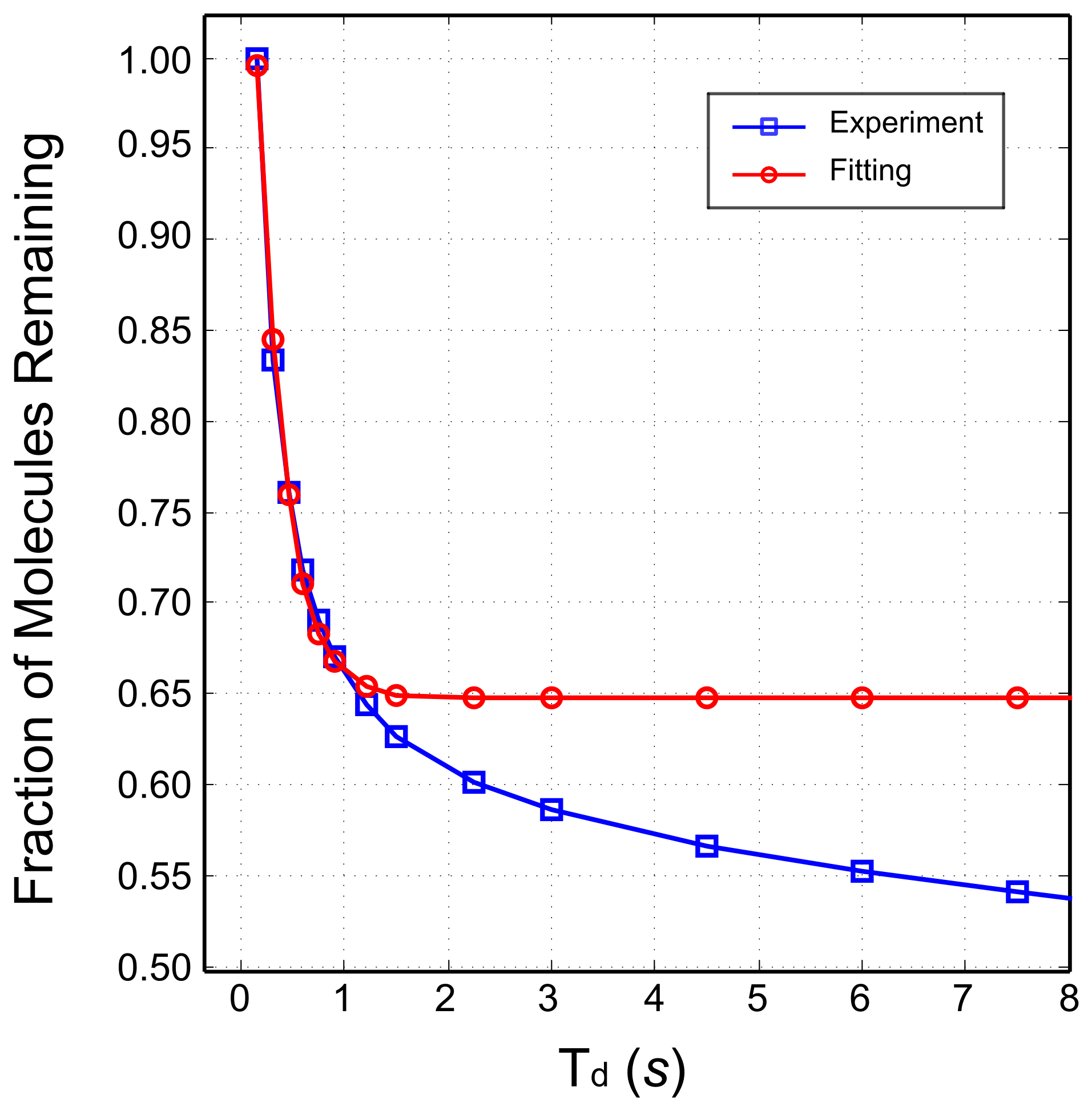

Supplement: Figure S4 — Estimating the dark state life time ( Toff ) of BiFC-PAmCherry1. We use a similar approach as described in Annibale et al. (ref 22) to estimate the T off of PAmCherry1 reconstituted by BiFC. Briefly, the total number of molecules in the final reconstructed PALM image is a function of maximum allowed dark period (T d). The greater T d is the smaller number of molecules remain in the final PALM image because more localization events are combined despite that they are separated by dark periods. This is reflected in the blue curve, where an initial, sharp decrease in the remaining fraction of molecules is followed by a second, slower decrease. The initial phase of the decrease is primarily due to the correction of molecular blinking, i.e., the molecules transiently entering dark states. As T d becomes much larger than Toff, emission events from different molecules residing in the same pixel start to get combined resulting in further decrease in the number of molecules. We found that the first 7 points (T d up to ∼1 s) gave the best fit to a single exponential (R = 0.996); from this fitting (red curve), we obtained T off ∼0.26±0.05 s. (TIF) [file pone.0100589.s004.tif]

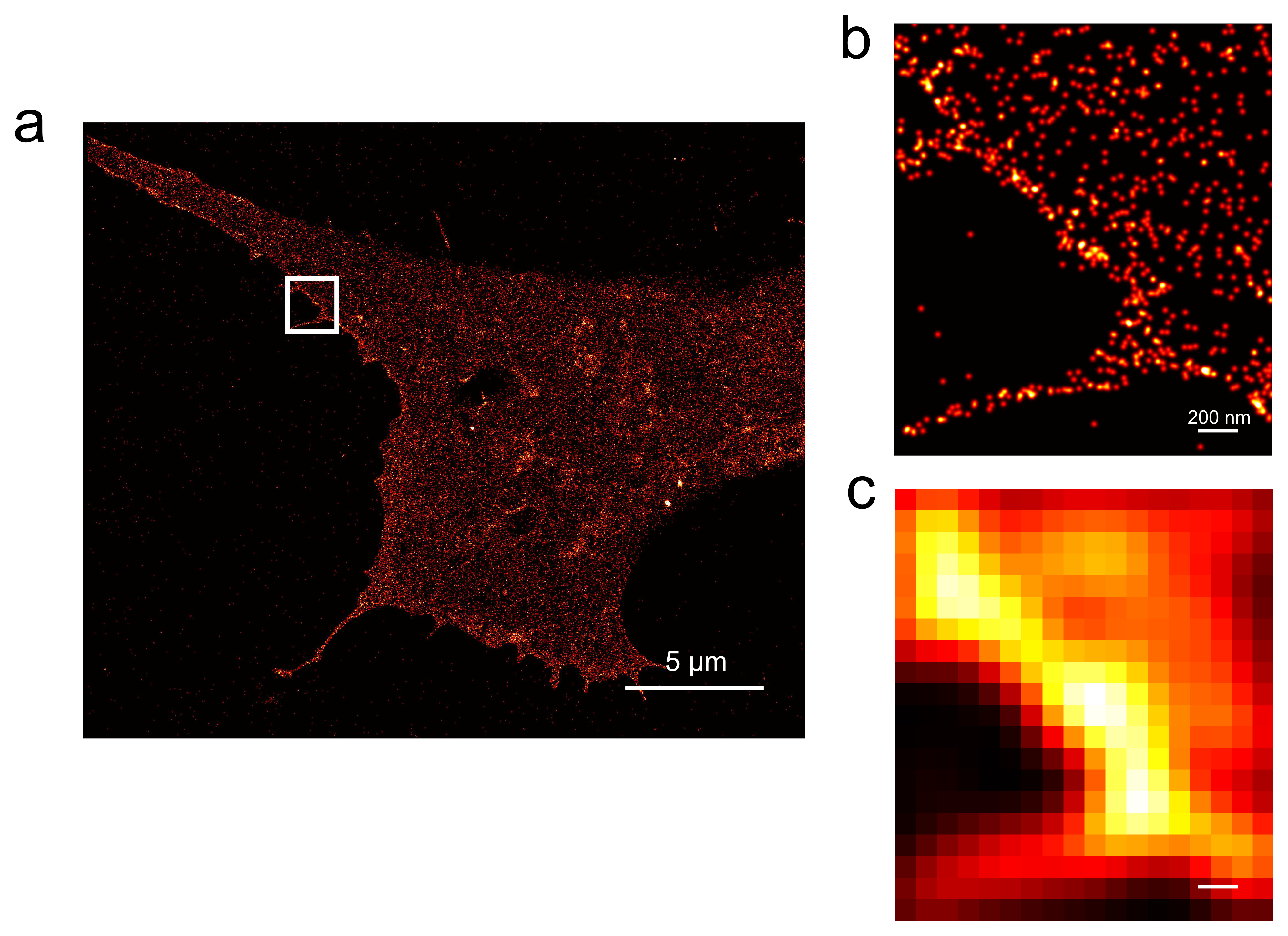

Supplement: Figure S5 — Superresolution imaging of DmrA/DmrC complex with BiFC-PALM. The BiFC configuration used was DmrA-RC/RN-DmrC, where RN and RC are PAmCherry1 fragments split at site 159. (a) PALM image of a cell expressing the BiFC pair; (b) Zoomed-in view of the boxed area in (a); (c) Low-resolution representation of (b). (TIF) [file pone.0100589.s005.tif]

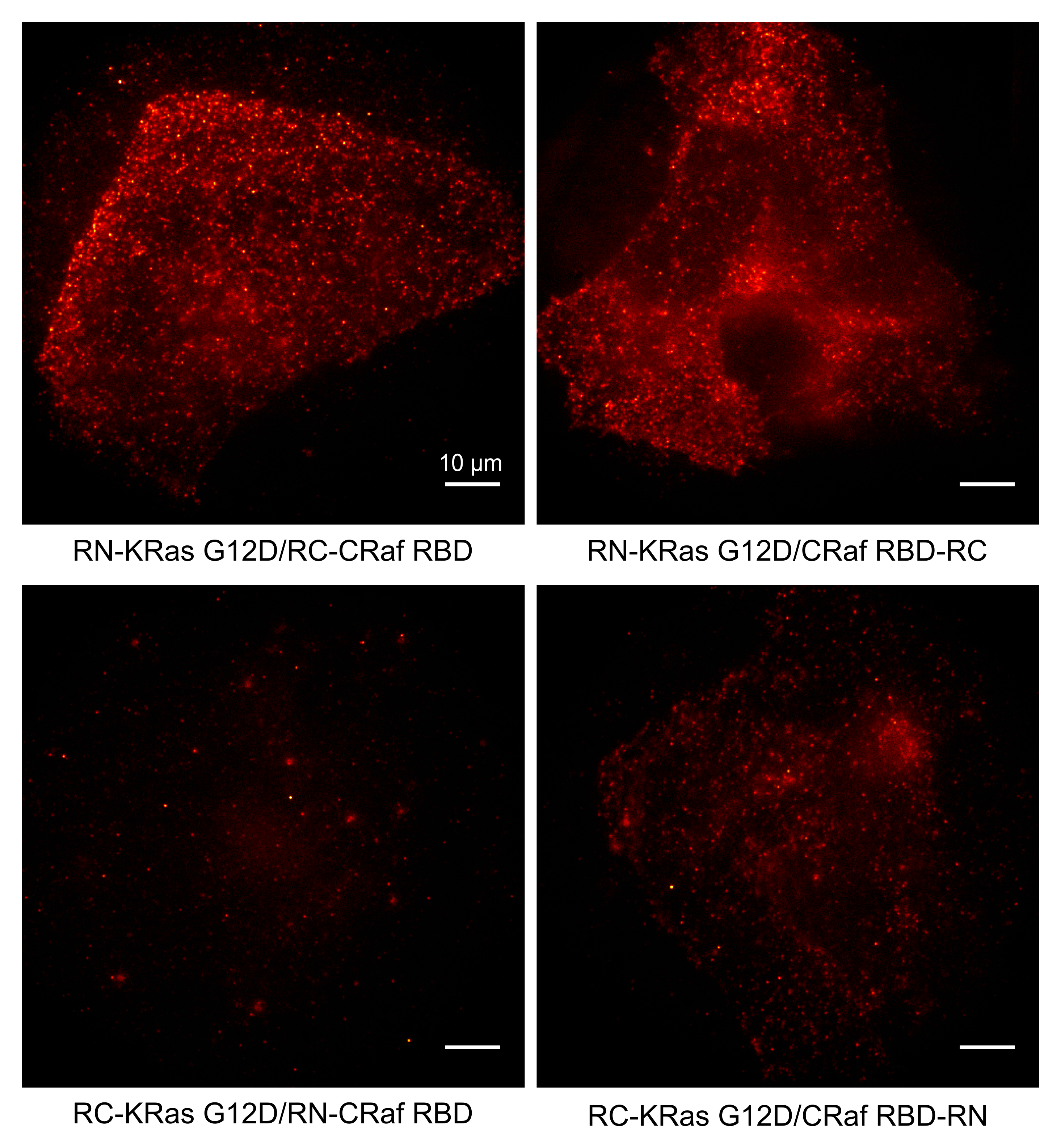

Supplement: Figure S6 — PAmCherry1 BiFC to visualize KRas G12D/CRaf RBD interaction. TIRF images for the four BiFC configurations between KRas G12D and CRaf RBD, each fused with PAmCherry1 fragments RN or RC (split at site 159). U2OS cell lines stably expressing RN-KRas G12D (top panels) or RC-KRas G12D (bottom panels) were generated. RC-CRaf RBD or CRaf RBD-RC was then introduced into either cell line via lentiviral infection ∼24 hours prior to imaging. RN-KRas G12D/CRaf RBD-RC (upper right) was used for Fig. 3B, D and E. (TIF) [file pone.0100589.s006.tif]

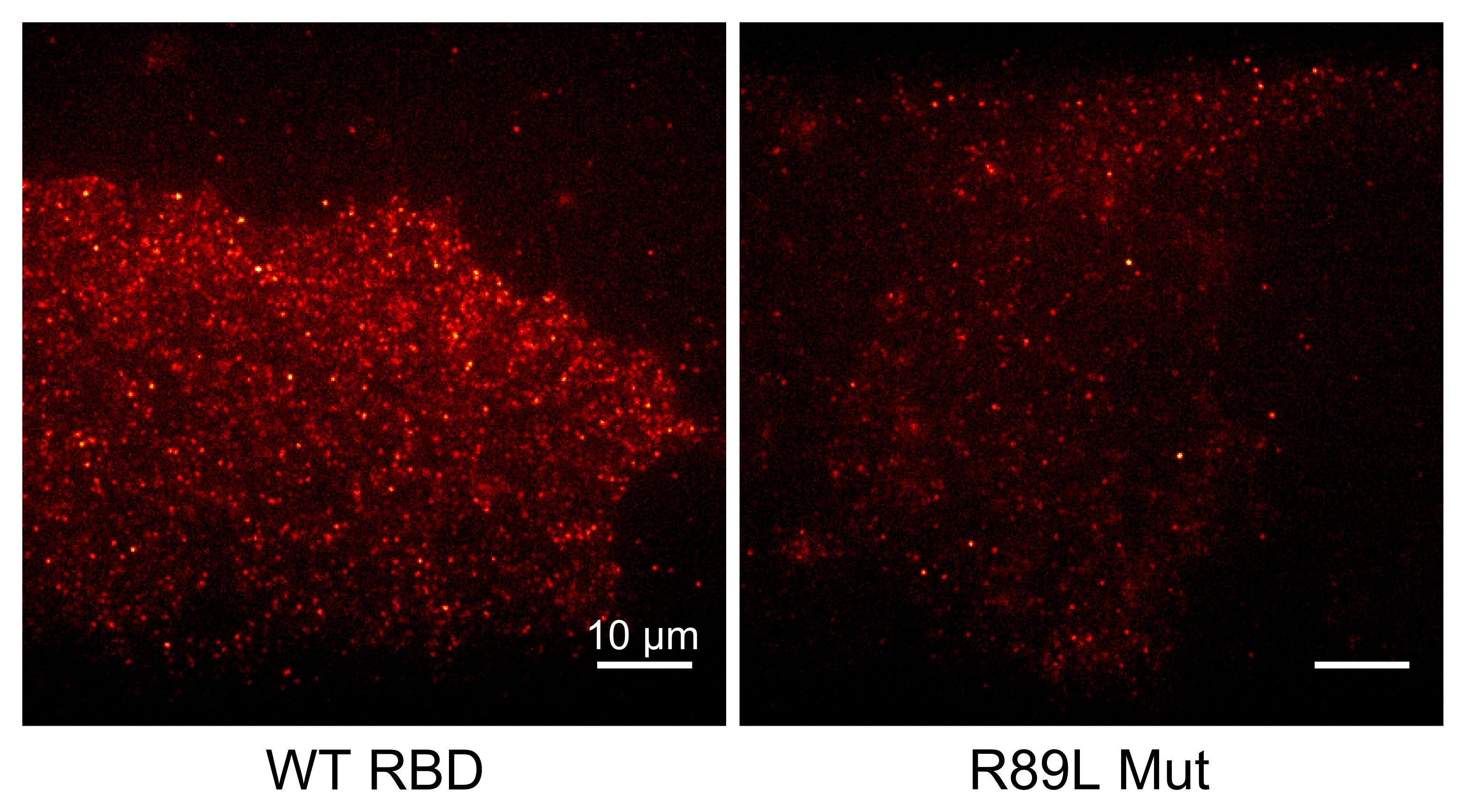

Supplement: Figure S7 — Effect of the RBD R89L mutation on BiFC of RN-KRas G12D and CRaf RBD-RC. U2OS cells stably expressing RN-KRas G12D were infected with lentivirus bearing wildtype CRaf RBD-RC (left) or the CRaf RBD R89L-RC mutant (right) and fixed ∼24 hours post infection. (TIF) [file pone.0100589.s007.tif]

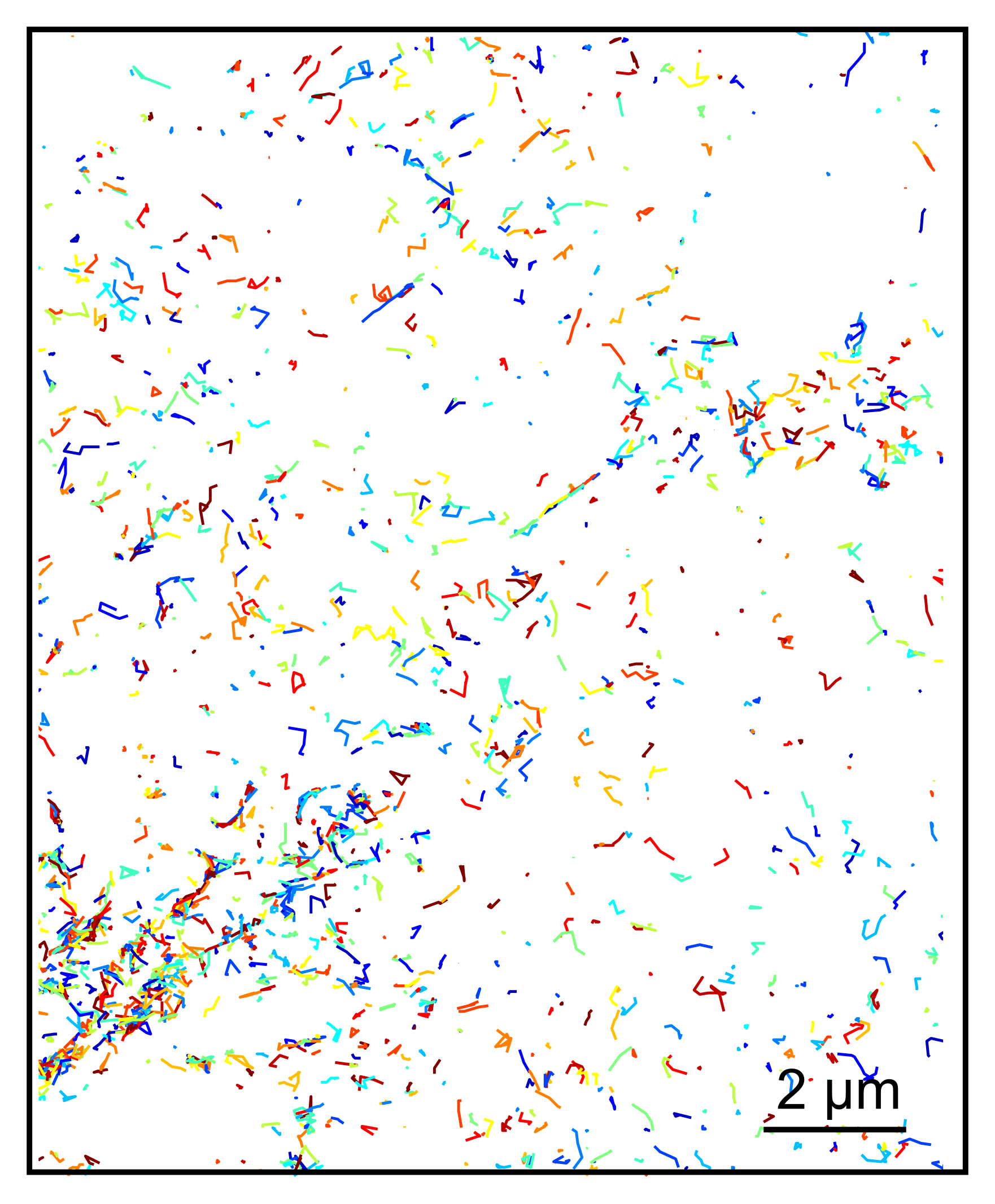

Supplement: Figure S8 — Single molecule tracking of individual KRas G12D/CRaf RBD complexes. Live U2OS cells expressing RN-KRas G12D and CRaf RBD-RC were imaged with smt-PALM at 37°C and 50 ms time resolution. Individual molecules were localized and the diffusion trajectories were inferred from the locations of the same molecule in consecutive frames. The trajectories are randomly color-coded for easy distinction. (TIF) [file pone.0100589.s008.tif]
