# Supplementary material for: Photoactivated Localization Microscopy with Bimolecular Fluorescence Complementation (BiFC-PALM) for Nanoscale Imaging of Protein-Protein Interactions in Cells
Source: PLoS One. 2014 Jun 25;9(6):e100589. doi: 10.1371/journal.pone.0100589 (PMC4070983; doi:10.1371/journal.pone.0100589)
Supplement: Table S1 — PCR Primers used in this study. (DOCX) [file pone.0100589.s009.docx]

| PAmCherry_160-236-RKD R | CATGGTACCGAGCTCCTGCAGC |
| --- | --- |
| PAmCherry_160-236-RKD F | GAGCTCGGTACCATGGGCGCCCTGAAGGGCGA |
| Ras-PAmCherry_1-159 F | ATGTACCCCGAGGACTAAAAGGGTGGGCGCGCCGA |
| Ras-PAmCherry_1-159 R | GTCCTCGGGGTACATCCGCTC |
| MCS_IF_Fwd | CTAAAAGGGTGGGCGCGCC |
| N-MCS_Rev | TGAAGGGGGCGGCCG |
| L-N-MCS Fwd | GGTGGAGGTGGAAGTCCAAGCTTCTCGAGCTGCAGGAGC |
| PAmCh1-159-L Rev | ACTACCTCCACCTCCGTCCTGGGGGTA |
| L-ACC_PAmCh1-159 Fwd | GGTGGAGGTGGAAGTACCATGGTGAGCAAGGGCGAGGAG |
| L-PAmCh1-159 Fwd | GGTGGAGGTGGAAGTATGGTGAGCAAGGGCGAGGAG |
| DmrA Fwd | CGGCCGCCCCCTTCACCATGGGGAGTAGCAAGAGCAAGCC |
| DmrA-L Rev | ACTTCCACCTCCACCACTACCTCCACCTCCTTCCAGTTTTAGAAGCTCCACATCGA |
| L-DmrC Fwd | GGAGGTGGAGGTAGTGGTGGAGGTGGAAGTATGGCTTCTAGAATCCTCTGGCATGA |
| DmrC Rev | CGCCCACCCTTTTAGGATCCGAACTTTGAGATTCGTCGGAACACATGA |
| PAmCh160-236-L Rev | ACTACCTCCACCTCCCTTGTACAGCTCGTCCATGCCG |
| DmrC-L Rev | ACTTCCACCTCCACCACTACCTCCACCTCCCTTTGAGATTCGTCGGAACACATGA |
| L-PAmCh160-236 Fwd | GGTGGAGGTGGAAGTGGCGCCCTGAAGGGCGA |
| PAmCh1-159-L Rev | ACTACCTCCACCTCCGTCCTCGGGGTACATCCGCTC |
| DmrA-L-R161-236 F | GGTGGAAGTGCCCTGAAGGGCGAGGTCAAGCCAAGGGT |
| DmrA-L-R161-236 R | CTTCAGGGCACTTCCACCTCCACCACTACCTCCACCTCCTTC |
| DmrA-L-R159-236 F | TGGTGGAGGTGGAAGTGACGGCGCCCTGAAGGGCGA |
| DmrA-L-R159-236 R | GTCACTTCCACCTCCACCACTACCTCCACCTCCTTCCAGTTTTAGAA |
| R1-158-L-DmrC F | TACCCCGAGGGAGGTGGAGGTAGTGGTGGAGGTGGAAGT |
| R1-158-L-DmrC R | CACCTCCCTCGGGGTACATCCGCTCGGAGAGGGC |
| R1-160-L-DmrC F | GTACCCCGAGGACGGCGGAGGTGGAGGTAGTGGTGGAGG |
| R1-160-L-DmrC R | GCCGTCCTCGGGGTACATCCGCTCGGAGAGGGCCT |
| L-Ras Fwd | GGTGGAGGTGGAAGTACTGAATATAAACTTGTGGTAGTTGGAGCTGA |
| (L-)Ras(pENTR) Rev | GCGCCCACCCTTTTAGGATCC |
| (pENTR)FP-L-XC Fwd | TAAAAGGGTGGGCGCGCC |
| FP-L-XC Rev | ACTTCCACCTCCACCACTACCTCCA |
| RBD-L Fwd | CGGCCGCCCCCTTCACCATGCCTTCTAAGACAAGCAACACTATCCGTG |
| RBD-L Rev | ACTACCTCCACCTCCCAGGAAATCTACTTGAAGTTCTTCTCCAATC |
| NX-L-FP (Fwd) | GGAGGTGGAGGTAGTGGTGGAGG |
| L-RBD Fwd | GGTGGAGGTGGAAGTCCTTCTAAGACAAGCAACACTATCCGTG |
| (L-)RBD(pENTR) Rev | GCGCCCACCCTTTTACAGGAA |
| L-RBD Rev2 | GCGCCCACCCTTTTACAGGAAATCTACTTGAAGTTCTTCTCCAATC |
| RBD R89L Fwd | CTGGGCCTGCAACCAGAGTGCTG |
| RBD R89L Rev | TGGTTGCAGGCCCAGCACCTTGAGTGCTTTCATAAGGCAGTC |
| N-myr remove IF F | GCCCCCTTCACCATGTCTAGAGGAGTGCAGGTGGAAACCA |
| N-myr remove IF R | CATGGTGAAGGGGGCGGC |
| RC174 IF F | GGTGGAGGTGGAAGTGGCGGCCACTACGACGCTG |
| RC174 IF R | ACTACCTCCACCTCCGTCCTTCAGCTTGACCCTTGGCT |
